# Mass Casualty Decontamination in a Chemical or Radiological/ Nuclear Incident: Further Guiding Principles

**DOI:** 10.1371/currents.dis.569a83b893759346e511a070cb900d52

**Published:** 2016-09-15

**Authors:** Holly Carter, Richard Amlôt, Richard Williams, G. James Rubin, John Drury

**Affiliations:** Emergency Response Department, Public Health England, Salisbury, UK; Emergency Response Department, Public Health England, Salisbury, UK; Welsh Institute for Health and Social Care, University of South Wales, Pontypridd, Wales; Institute of Psychiatry, King's College London, London, UK; School of Psychology, University of Sussex, Brighton, UK

## Abstract

This short report presents a response to an article written by Cibulsky et al. (2016). The paper by Cibulsky et al. presents a useful and timely overview of the evidence surrounding the technical and operational aspects of mass casualty decontamination. It identifies three priority targets for future research, the third of which is how casualties' needs can be met in ways that best support compliance with and effectiveness of casualty decontamination. While further investigation into behavioural, communication and privacy issues during mass decontamination is warranted, there is now a substantial body of research in this area which is not considered in detail in the succinct summary provided by Cibulsky et al. (2016). In this short report, we summarise the available evidence around likely public behaviour during mass decontamination, effective communication strategies, and potential issues resulting from a lack of privacy. Our intention is to help further focus the research needs in this area and highlight topics on which more research is needed.

## Introduction

This short report presents a response to the article written by Cibulsky et al. (2016) 1 ‘Mass casualty decontamination in a chemical or radiological/ nuclear incident with external contamination: guiding principles and research needs’. The paper by Cibulsky et al. presents a useful and timely overview of the evidence surrounding the technical and operational aspects of mass casualty decontamination. It identifies three priority targets for future research: operational analysis to determine the best ways to integrate casualty decontamination into the incident response and coordinate it with medical evaluation and treatment; comparative study of the efficacy of various decontamination methods and their potential adverse effects; and behavioural, communication and privacy issues, including what casualties and community members need during an incident that requires mass decontamination, and how their needs can be met in ways that best support compliance with and effectiveness of casualty decontamination.

We agree that each of these areas is a priority for future research. However, while further investigation into behavioural, communication and privacy issues during mass decontamination is warranted, there is now a substantial body of research in this area which is not considered in detail in the succinct summary provided by Cibulsky et al. (2016) 1. In this short report, we summarise the available evidence around likely public behaviour during mass decontamination, effective communication strategies, and potential issues resulting from a lack of privacy. Our intention is to help further focus the research needs in this area and highlight topics on which more research is needed.

## Behavioural (likely public behaviour)

Traditionally, planning and policy for mass decontamination has focused on the technical aspects of mass decontamination, such as developing and testing decontamination equipment, with little consideration of likely public behaviour. Where likely public behaviour has been considered in planning for incidents involving mass decontamination, there has been a reliance on common myths about crowd behaviour, such as inherent public disorder and mass panic 2. However, over 50 years of research has shown that panic occurs very rarely during mass emergencies and disasters, and that people are much more likely to behave in a helpful and cooperative way 3 ^,^ 4 ^,^ 5 ^,^ 6 ^,^ 7. This reliance on assumptions about panic has resulted in little attempt to develop strategies for communicating with members of the public during decontamination; if people are going to behave in an irrational way then there won’t be any point in trying to communicate with them. Indeed, research suggests that assumptions about panic may lead to information being withheld from members of the public, which may actually create the very disorder 8 ^,^ 9 and non-compliance 10 which authorities are hoping to prevent.

Evidence from small-scale incidents involving decontamination shows that those people who are affected may refuse to comply with decontamination procedures if they are not provided with sufficient information about why decontamination is necessary, and what the process involves 2. Findings from large-scale field exercises and field trials involving mass decontamination suggest that, if sufficient information is provided and the people involved believe that responders are being open with them, members of the public are likely to be willing to comply with decontamination 11 ^,^ 12, and also willing to help others to undergo decontamination 12 ^,^ 13.

Overall, these research studies show that public behaviour is likely to be contingent on the way in which emergency responders manage the incident. Emergency responders who communicate effectively with members of the public and show respect for their needs change the relationships and that, consequently, results in more positive outcomes from the incident, in terms of reduced public anxiety and increased public compliance and cooperation. Thus, if managed well, members of the public can actually be an asset to emergency responders in their attempts to successfully manage the incident.

## Communication and privacy

Effective communication is essential during mass decontamination – failure to communicate effectively can result in reduced public compliance and cooperation, increased confusion, and even attempts to challenge responders’ authority. Several research studies have examined how different perceptions of responder communication can affect public compliance and cooperation, as well as levels of public anxiety, during mass decontamination 11 ^,^ 14 ^,^ 15.

A recent mass decontamination field experiment specifically tested three different responder communication strategies, in order to try to identify what makes some communication strategies more effective than others 12. Results from this study revealed that, for a communication strategy to be effective during mass decontamination, it should include open and honest information about the nature of the incident, health-focused explanations about the importance of decontamination, and sufficient practical information to enable those people who are affected to successfully undergo decontamination. Crucially, the inclusion of an effective communication strategy not only resulted in more positive psychological outcomes for those affected, it also resulted in improved speed and efficiency of decontamination on objective measures 12. Therefore, this could save lives during a real incident.

As well as the provision of effective responder communication, another key issue during mass decontamination will be whether members of the public feel they have sufficient privacy 16 ^,^ 17 ^,^ 18. It is crucial that emergency responders show that they are doing what they can to respect public needs for privacy and modesty; failure to do so can result in reduced public compliance and cooperation 2 ^,^ 12 ^,^ 13 ^,^ 14 ^,^ 19.

Several research studies have examined the mechanisms underlying the relationships between effective responder communication, sufficient privacy, and positive outcomes during mass decontamination 12 ^,^ 14 ^,^ 20. Findings show that effective communication from emergency responders and the provision of sufficient privacy are crucial because they enhance public perceptions that responders are behaving in a legitimate way. Enhanced perceptions of responder legitimacy facilitate increased identification between members of the public and emergency responders, as well as amongst members of the public, and it is this identification which leads members of the public to actively engage with the decontamination process.

The findings from these research studies have been used to generate recommendations for emergency responders when managing incidents involving mass decontamination 15, and so far have been included in US decontamination guidance documents for emergency responders 21 ^,^ 22 ^,^ 23.

## Conclusion

This research programme can be used to inform best practice for managing casualties during mass decontamination. However, as noted in Cibulsky et al. (2016)^1^, there is a need for further research in this area to optimise casualty management strategies for mass decontamination. Ongoing work related to the development of optimal management strategies for mass decontamination is being carried out as part of the NIHR Emergency Preparedness and Response Health Protection Research Unit (EPR HPRU) (http://epr.hpru.nihr.ac.uk/). This research includes a qualitative study of factors which affect perceived public acceptability of different decontamination methods, part of which involves testing the effect of different responder management strategies on perceived public acceptability of mass decontamination. The research also examines cultural factors which may affect public willingness to comply with different decontamination methods. Further research could include analysis of data from real incidents involving decontamination, as well as new field studies and exercises testing different responder management strategies. In particular, future research should consider practical aspects of communication, such as how best to deliver information to members of the public during the decontamination process, and also how best to protect casualties’ privacy and so promote compliance with the decontamination process.

## Competing Interests

The authors have declared that no competing interests exist.

## Correspondence

Holly Carter: holly.carter@phe.gov.uk
